# Preparation and Properties of Carbon Fiber/Carbon Nanotube Wet-Laid Composites

**DOI:** 10.3390/polym11101597

**Published:** 2019-09-30

**Authors:** Suhyun Lee, Kwangduk Ko, Jiho Youk, Daeyoung Lim, Wonyoung Jeong

**Affiliations:** 1 Human Convergence Technology Group, Korea Institute of Industrial Technology, Ansan 15588, Korea; suhyun14@kitech.re.kr (S.L.); gdgdgd1207@naver.com (K.K.); zoro1967@kitech.re.kr (D.L.); wyjeong@kitech.re.kr (W.J.); 2 Department of Chemical Engineering, Inha University, Incheon 22212, Korea; youk@inha.ac.kr

**Keywords:** carbon fiber-reinforced composites, carbon nanotubes, polyamide 6, wet-laid process, electrically conductive materials

## Abstract

In this study, carbon nanotubes (CNTs) were introduced into carbon fiber (CF) wet-laid composites as functional nano-fillers to fabricate multi-functional composites with improved mechanical, electrical, and thermal properties. It was considered that the wet-laid process was most suitable in order to introduce filler into brittle and rigid carbon fiber substrates, and we established the conditions of the process that could impart dispersibility and bonding between the fibers. We introduced polyamide 6 (PA6) short fiber, which is the same polymeric material as the stacking film, into carbon fiber and CNT mixture to enhance the binding interactions between carbon fiber and CNTs. Various types of CNT-reinforced carbon fiber wet-laid composites with PA6 short fibers were prepared, and the morphology, mechanical and electrical properties of the composites were estimated. As CNT was added to the carbon fiber nonwoven, the electrical conductivity increased by 500% but the tensile strength decreased slightly. By introducing short fibers of the same material as the matrix between CNT–CF wet-laid nonwovens, it was possible to find optimum conditions to increase the electrical conductivity while maintaining mechanical properties.

## 1. Introduction

Among the fiber-reinforced composites, carbon fiber (CF)-reinforced composites are used widely in the aerospace, automobile, sports, medical instrument, and construction industries as well as in the military because of their high mechanical properties, impact resistance, thermal resistance, chemical stability, and lightweight nature [1,2,3,4,5,6,7,8,9,10,11].

Generally, the properties of carbon fiber-reinforced composites are determined not only by the carbon fiber content, fiber length, fiber orientation, and fiber matrix adhesion, but also by the inherent characteristics of the polymer matrix [6,10,12]. Carbon fiber-reinforced composites exhibit excellent in-plane properties, but they typically show poor out-of-plane performance that is dominated by the polymer matrix [8]. Without a surface treatment with a polymer matrix, carbon fibers have poor wettability and adsorption with most polymers because they have a non-polar surface and are comprised of highly crystallized, graphitic basal planes with inert structures [6]. Among the matrix polymers available, polyamide 6 (PA6) is considered a good candidate as a carbon fiber-reinforced composite matrix, mainly because of its various mechanical properties, low cost, and easy handling [5,6,13,14]. One impregnation technique for obtaining polyamide continuous reinforcement composites is the use of interfacial polycondensation where the reinforcement is impregnated as it is passed and pulled across the polyamide reaction chamber. Another technique is impregnation by hot press molding, where the reinforcement is impregnated by a polymer matrix under heat and pressure [5].

Carbon nanotubes (CNTs) are strong nanomaterials with high mechanical, thermal, and electrical properties [7,8,9,10,15]. CNTs have recently been considered for use as reinforcements in advanced composites because of their high strength and conductivity compared to existing fibers [16,17,18,19,20,21,22]. Chou et al. [8] reported the potential of CNT–CF hybridization. They grafted CNTs on the carbon fiber using a direct growth method and measured the interfacial shear strength in single-fiber composites. The interfacial shear strength was improved considerably because of the increased surface area of the CNT-grafted carbon fiber. Zhao et al. [17] presented a synthesis approach by grafting CNTs onto carbon fibers via a layer-by-layer method. They demonstrated that the presence of CNTs improves the interfacial properties and mechanical interlocking between the carbon fibers and epoxy matrix effectively. Shin et al. [19] also incorporated high concentrations of CNTs into carbon fiber-reinforced epoxy composites and suggested that the insertion of a CNT mat between the carbon fiber fabric leads to the highest electrical conductivity and thermal conductivity. In addition, several studies have focused on developing an effective CNT–CF hybridization method, such as the growth of CNTs on the carbon fiber surface by chemical vapor deposition (CVD) [7,23], coating of CNTs on the carbon fiber surface by electrophoresis [8,20] or spraying [9] and CNTs dispersed in a matrix [10,18,19]. On the other hand, CNTs are difficult to disperse homogeneously and good adhesion between CNTs, carbon fiber, and polymer matrix is problematic because CNTs tend to agglomerate and entangle with each other due to their strong Van der Waals bonds [10,24,25]. Therefore, it is important to improve the dispersion of CNTs in carbon fiber-reinforced composites.

This study developed carbon fiber-reinforced composites with improved mechanical properties and electrical conductivity using CNTs. In particular, the present study suggests that by adding PA6 fibers to the CNT–CF mixture, which was prepared for nonwoven fabric, the interfacial bonding strength of the CNT–CF-nonwoven fabric is enhanced by PA6 fiber melting through the film stacking process. For this purpose, CF, CNTs, and PA6 fiber were dispersed in water and a wet-laying process was used to produce the CNT–CF-nonwoven fabric. The fabricated CNT–CF-nonwoven composite was infiltrated with a PA6 film by a hot press molding process for film stacking to improve the toughness, environmental resistance, and usability. The mechanical properties, electrical conductivity, and thermal properties were then observed and compared according to the contents of CNTs and PA6 fiber.

## 2. Materials and Methods

### 2.1. Materials

Carbon fiber (T700; 12K, 800TEX) was purchased from Toray (Tokyo, Japan) and cut into 10-mm-long pieces using a Fidocut machine (Schmidt & Heinzmann, Bruchsal, Germany). CNTs (Hanwha Chemical Co., Ltd., Seoul, Korea) were used multi-wall carbon nanotubes with a purity of 97%, 10–15 nm in diameter, and 180–200 μm in length. Kolliphor P188 as a surfactant was purchased from Sigma-Aldrich (St. Louis, MO, USA). Polyvinyl alcohol (PVA) from Kurary (Tokyo, Japan) was prepared as a binder. Polyamide 6 (PA6) fiber, 0.4 mm in length of 0.4 denier, and films, 58 g/m^2^ in weight and 0.06 mm in thickness, were obtained from Sam Young Chemical (Seoul, Korea). All reagents were used as received.

### 2.2. Fabrication of CNT–CF-Nonwoven

**Figure 1:**
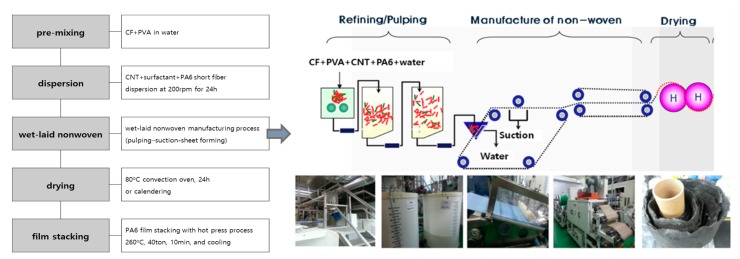
Schematic diagram of the process: CF, carbon fiber; CNT, carbon nanotube; PA6, polyamide 6; PVA, polyvinyl alcohol.

Two grams of carbon fiber (0.1 wt %) and 0.2 g of PVA (0.01 wt %) were dispersed in 2 L of water. CNTs (0–20 wt % with respect to carbon fiber loading), PA6 staple fiber (0, 50, and 100 wt % with respect to CNTs loading), and surfactant (0.1 wt % with respect to water) were then added in sequence to the mixture and stirred at 200 rpm for 24 h. A small amount of surfactant and PVA were added to disperse the carbon fibers and CNTs and to provide the minimum binding force for web formation. The CNT–CF-nonwoven fabric was then made using a wet laying process, as shown in Figure 1. The wet laying process is environmentally friendly, using water as a solvent; the quantity of carbon fiber and CNTs added can be controlled and all thermoplastic polymers can be applied. After the fibers were disintegrated and dissociated through the pulper, the web was manufactured through a wet-laid process. Finally, the nonwoven fabric samples were fabricated through the drying process in an oven for 24 h.

### 2.3. Film Stacking by Hot Press Molding

A film stacking process using hot press molding was carried out in a steel mold. First, the CNT–CF-nonwoven fabric was heated to 260 °C and held at this temperature for 20 min. Subsequently, it was pressed under 40 ton of pressure, and held for 10 min with PA6 film. The mold was then cooled to 70 °C.

### 2.4. Characterization

The morphology of the CNT–CF-nonwoven composites was observed by field emission scanning electron microscopy (FE-SEM; SU8010, Hitachi, Tokyo, Japan). Prior to the examination, the sample surfaces were sputter-coated with platinum. The mechanical properties of the CNT–CF-nonwoven composites with various CNTs and PA6 fiber contents were characterized according to ASTM D638 using a universal testing machine (Instron 3343, Norwood, MA, USA). The sample size was 100 mm × 20 mm for testing. The gauge length and crosshead speed were 60 mm and 10 mm/min, respectively. The electrical conductivities of the CNT–CF-nonwoven composites were characterized using a 4-point probe machine (CMT-100S, Advanced instrument technology, Suwon, Korea). Thermogravimetric analysis (TGA; Q500, TA instrument, New Castle, DE, USA) was performed to assess the degradation temperature and the weight fraction of the CNT–CF-nonwoven composites at a heating rate of 10 °C/min in a nitrogen atmosphere.

## 3. Results

### 3.1. Analysis of the Structure of the CNT–CF-Nonwoven Composites

CNT–CF-nonwoven composites with different structures were prepared from carbon fibers and CNTs to examine the effects of the mechanical properties and electrical conductivity according to the manufacturing process. 

**Figure 2:**
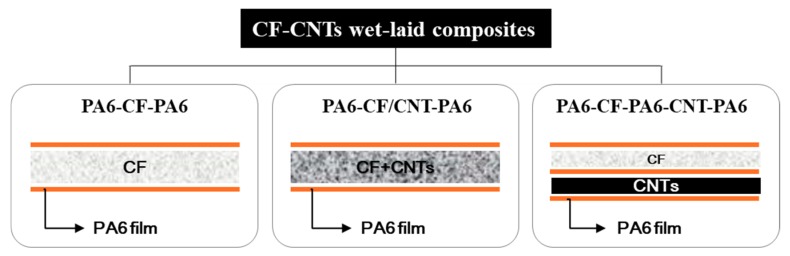
Schematic of structure of the CNT–CF-nonwoven samples.

Figure 2 presents a schematic diagram of the CNT–CF-nonwoven samples. The structure of the CNT–CF-nonwoven composites was examined using two methods. One was making a single layer of nonwoven fabric by mixing carbon fibers and CNTs, simultaneously. The other was making each layer of nonwoven fabric by carbon fibers and CNTs, separately, and integrating them with a PA6 film. For this preliminary experiment, CF (2 g) and CNT (1 g) were dispersed in water, and then prepared in separate layers or in a mixed layer. The prepared CF and CNT web was completed as a composite using a PA6 film stacking process.

Table 1 lists the properties of CNT–CF-nonwoven composites according to the structure. The thickness and weight of the PA6–CF/CNT–PA6 and PA6–CF–PA6–CNT–PA6 samples were higher than those of PA6–CF–PA6. These results were attributed to CNT, which has a large volume fraction with a high aspect ratio [16].

The electrical conductivity of the carbon fiber-reinforced composites was low because the PA6 film in the composites is an insulator that interferes with the flow of electrons. The introduction of CNTs into the carbon fiber-reinforced composites was expected to enhance the electrical conductivity because they have outstanding electrical properties and can promote the formation of an electrical network in the composites [8,19]. The conductivity of the CNT–CF-nonwoven samples was improved by the insertion of CNTs to 120.48 S/cm for PA6–CF/CNT–PA6 and 33.11 S/cm for PA6–CNT–PA6–CF–PA6. In particular, the conductivity of PA6–CF/CNT–PA6 composed of carbon fibers and CNTs as a single layer was improved significantly, compared to PA6–CNT–PA6–CF–PA6, which was composed of layers of carbon fibers and CNTs. This was attributed that the network of highly interconnected PA6–CF/CNT–PA6 benefits from the formation of conducting paths spanning the relatively large regions, as opposed to the PA6–CNT–PA6–CF–PA6, which are composed individual layers of carbon fibers and CNTs with a PA6 film layer [8].

On the other hand, in the case of the mechanical properties, the tensile strength and modulus decreased due to the CF/CNT mixture. The mechanical properties of the carbon fiber-reinforced composites depend mainly on the adhesion between the fibers and the matrix [6]. PA6–CF–PA6 and PA6–CNT–PA6–CF–PA6 showed similar tensile strength and modulus. This means that the adhesion force among the carbon fiber, CNTs and PA6 film interface is similar. On the other hand, the mechanical properties of PA6–CF/CNT–PA6 were drastically lower than those of the other samples. Several studies reported that the fabrication of carbon fiber and CNT composites is extremely difficult and imposes several limitations, such as an inhomogeneous distribution and poor bonding with the matrix [7,16]. 

From the results, these manufacturing methods limit the reinforcing strength because of the weak physical adsorption between the carbon fibers and CNTs. Therefore, in this study, PA6 fiber was introduced to the CNT–CF mixture for nonwoven composites to prevent the deterioration of the mechanical properties of composites and realize multi-functionality, such as electrical conductivity. PA6 is a semi-crystalline thermoplastic polymer generally used in many tribological applications because of the balance between its various mechanical properties and interfacial interaction [6]. By dispersing the PA6 short fiber in the CNT–CF-nonwoven fabric, the mechanical and electrical properties of the composites are expected to improve as the PA6 fiber melts inside the nonwoven composites and acts as a binder between the carbon fibers and CNTs through a film stacking process.

CNT–CF-nonwoven composites were manufactured according to various CNT and PA6 contents. Table 2 lists the final sample codes of each CNT–CF-nonwoven sample.

### 3.2. Morphology of CNT–CF-Nonwoven Composites According to the PA6 and CNT Contencts

**Figure 3:**
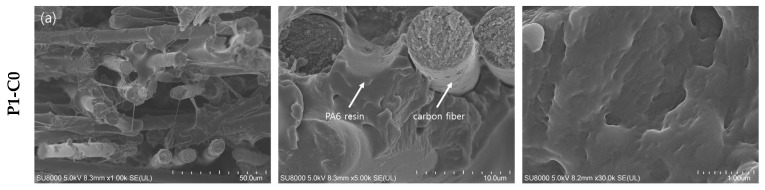

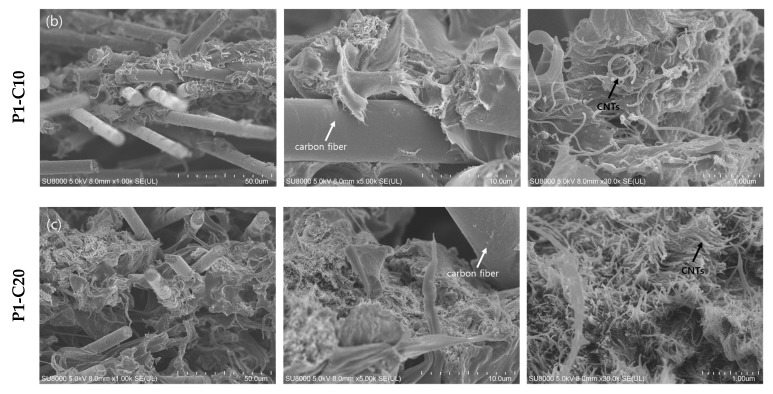
Cross section images of the CNT–CF-nonwoven composites with various CNT contents: (**a**) P1-C0; (**b**) P1-C10; and (**c**) P1-C20 with magnification ×1k, ×5k, and ×30k, respectively.

Figure 3 shows surface and cross section images of the CNT–CF-nonwoven according to various CNT contents without the PA6 fiber. In the cross-sectional image, it can be seen that CNTs are well distributed among the carbon fibers according to the CNT content. On the other hand, the surface images show that the PA6 film deposited by the film stacking process did not adhere uniformly to the surface of the CNT–CF-nonwoven composite as the CNT content was increased, and the formation of pores increased. This means that PA6, which can serve as a matrix between the carbon fibers and CNTs, did not penetrate into the CNT–CF nonwoven sufficiently. 

**Figure 4:**
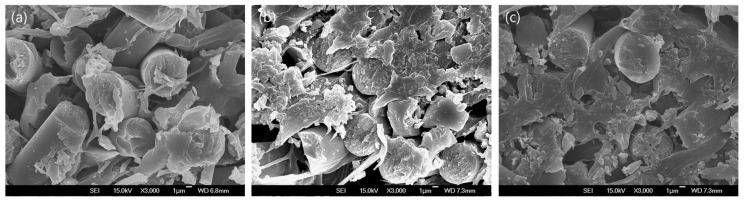
Surface and cross section images of the CNT–CF-nonwoven composites with various PA6 contents: (**a**) P1-C10; (**b**) P2-C10; and (**c**) P3-C10; with magnification ×30k.

The interfacial adhesion between fibers and polymer matrix plays an important role in improving the mechanical behavior of carbon fiber-reinforced composites [10]. Therefore, to strengthen the adhesion between the carbon fiber and CNTs with the polymer matrix, various contents of PA6 short fibers were introduced into the CNT–CF-nonwoven. Figure 4 shows the morphology of the CNT–CF-nonwoven samples according to the contents of PA fibers.

As shown in Figure 4, unlike the P1-C10 sample with no PA6 short fiber introduced, in P2-C10 and P3-C10, which contained the PA6 short fiber, the PA6 polymer was distributed evenly throughout the CNT–CF-nonwoven composites. This might be due to the homogeneous support of the carbon fibers resulting from the good dispersibility and interfacial interactions between the CNTs and PA6 matrix [10]. Some studies have indicated that the interfacial interaction mechanisms of CNTs, carbon fiber, and PA6 matrix can be ascribed mainly to three aspects: (i) the Van der Waals interaction among the CNTs; (ii) chemical reaction of CNTs with carbon fibers and the PA6 matrix; and (iii) mechanical interlocking between the CNTs and polymer matrix [17]. 

These results confirmed that the polymer matrix was formed inside the CNT–CF-nonwoven material by the introduction of PA6 short fibers through the wet-laid process.

### 3.3. Mechanical Properties of the CNT–CF-Nonwoven Composites

**Figure 5:**
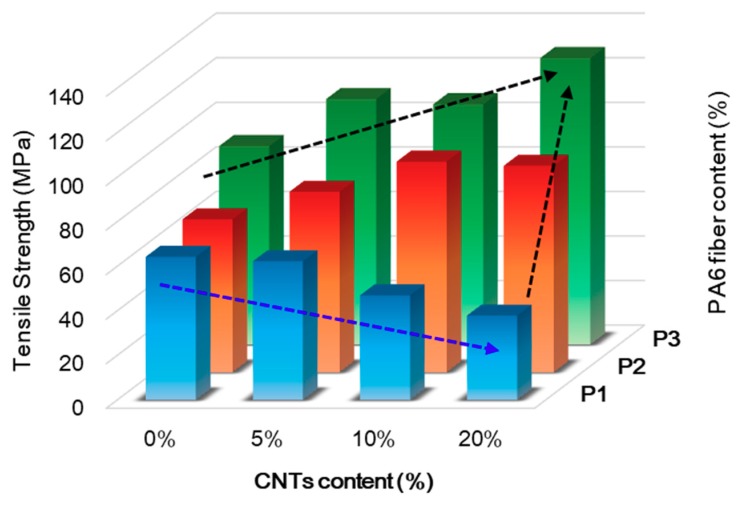
Mechanical properties of the CNT–CF-nonwoven composites.

The mechanical properties of the CNT–CF-nonwoven composites were examined by applying a tensile load. Figure 5 shows the tensile strength of the CNT–CF-nonwoven composites according to the CNTs and PA6 fiber contents. 

The tensile strength of the CNT–CF-nonwoven composite increased with increasing PA6 short fiber content. For the samples without CNTs, the tensile strengths were 64.24, 68.60, and 89.03 MPa for P1-C0, P2-C0, and P3-C0, respectively. This tendency was the same until up to 20 wt % of CNTs (37.84, 92.63, and 128.59 MPa for P1-C20, P2-C20, and P3-C20, respectively) because the PA6 short fiber reduced the empty space and facilitated load transfer in the composites. In addition, the mechanical properties of the carbon fiber-reinforced composites are closely related to the interfacial bonding between the carbon fiber surface and polymer matrix, which is relatively low because of the non-polar surface of the carbon fibers [13,26]. Carbon fibers have some chemical groups, such as carboxyl, epoxy, ether, and hydroxyl groups, which easily form chemical bonds with the amide group of the matrix PA6 [6]. Therefore, as the contents of PA6 increased, the chemical bonding between the carbon fiber and PA6 matrix became stronger due to the increased number of functional groups capable of chemical bonding with the carbon fiber, resulting in an increase in tensile strength.

On the other hand, in terms of the effects of CNTs, the tensile strength of P1, which was CNT–CF-nonwoven without a PA6 short fiber, decreased gradually with increasing CNT content. The tensile strengths were 64.24, 62.23, 46.91, and 37.84 MPa for P1-C0, P1-C5, P1-C10, and P1-C20, respectively. The reason for this result can be seen in the cross section images in Figure 3 and Figure 4. CNTs occupied the space between the carbon fibers in the nonwoven composite instead of PA6, which shows a lack of interaction between the carbon fiber and CNTs [12]. Therefore, the medium of facilitated load transfer was not present in the composites. This can also be explained by the increased stress build-up around the carbon fibers due to the introduction of CNTs on the composites. CNTs can act as defects and degrade certain properties, such as strength [27]. As a result, the tensile strength of the CNT–CF-nonwoven composite decreased with increasing CNT content.

In the case of composites containing both CNTs and PA6 short fiber, however, CNTs and PA6 had a positive effect on the tensile strength of the CNT–CF-nonwoven composites. As shown in Figure 5 for P2 and P3, the tensile strength of the CNT–CF-nonwoven composite increased with increasing CNT content. In addition, the tensile strength was improved further as the content of PA6 was increased. The maximum value was achieved by the introduction of 20 wt % CNTs and 100 wt % PA6 short fibers, and the strength increased from 64.24 MPa to 128.59 MPa. Bekyarova et al. [8] reported that the addition of CNTs plays a significant role in reinforcing the polymer matrix. According to their research, the introduction of ~0.25% CNTs in the CF/epoxy composites enhanced the interlaminar shear strength by 27%. The incorporation of CNTs at the composite interface can increase the specific surface area of the reinforcement filler significantly, which results in an improvement in the Van der Waals interactions at the composite interface, such as PA6 [18]. Therefore, in this study, the surface area that can be conjugated with the polymer matrix increased with the introduction of CNTs into the composite material. Because the PA6 polymer formed a strong chemical bond between the CNTs and carbon fiber, the interfacial interaction was enhanced considerably, and the tensile strength was improved [26]. In addition, as CNTs are inserted to the carbon fiber-reinforced nonwoven composites with PA6 fibers, the mechanical stiffness of the samples can be increased due to the entangled CNTs, resulting in an increased tensile strength of the CNT–CF-nonwoven composites [9]. 

Overall, the presence of CNTs bound the carbon fibers and polymer matrix together, which enhanced load transfer at the interface [20,23].

### 3.4. Electrical Properties of the CNT–CF-Nonwoven Composites

**Figure 6:**
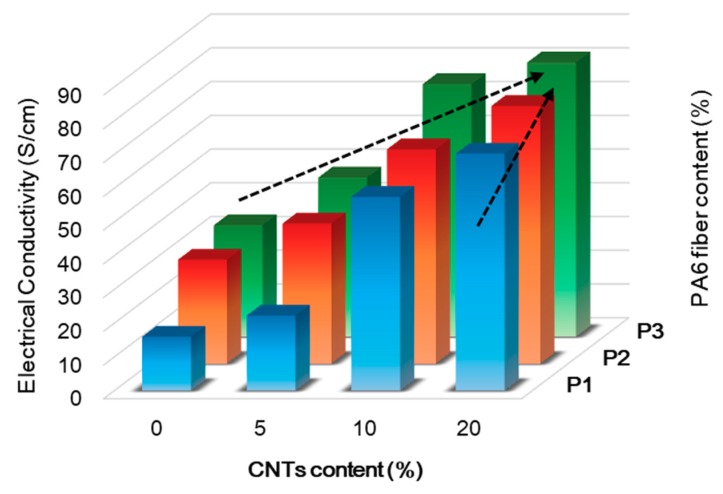
Electrical properties of the CNT–CF-nonwoven composites.

The electrical conductivity was examined by measuring the surface resistance using a 4-point probe system. The electrical conductivity was affected by the carbon fiber and CNT structure quality within the matrix, contents, dispersion, and the compatibility among the carbon fiber, CNTs, and the polymer matrix [24]. Figure 6 shows the electrical conductivity of the CNT–CF-nonwoven composites according to the CNTs and PA6 fiber contents. 

As in P1-C0, the electrical conductivity of pure carbon fiber nonwoven was 15.97 S/cm. The electrical conductivity increased to 32.99 S/cm with increasing PA6 short fiber content, which was attributed to the binding effect of the PA6 matrix in forming a channel for ion transfer between the carbon fibers.

The introduction of CNTs in the carbon fiber-reinforced composites resulted in significant improvement of the electrical conductivity of the CNT–CF-nonwoven composites as a result of the reinforcement of the electron transport channels [8]. The electrical conductivities of the CNT–CF-nonwoven according to the CNT content were 22.24 S/cm, 57.43 S/cm, and 70.23 S/cm for P1-C5, P1-C10, and P1-C20, respectively. The high electrical conductivity was attributed to the formation of excellent conductive CNT networks in the carbon fiber nonwoven composites. CNTs became a perfect auxiliary conductive network before the formation of the main conductive framework of the carbon fibers. In addition, the unique electrical properties of the CNTs also enhanced the conductivity of the carbon fiber-reinforced composites. Moreover, the CNTs provide free charges in the surroundings because of their specific surface area and low surface energy [21]. Therefore, as the CNT content in the nonwoven composites increases, the CNTs strengthen the role of the conductive bridge for electron flow inside of the composite, thereby improving the electrical conductivity. 

The electrical conductivity of the CNT–CF-nonwoven composites containing both CNT and PA6 short fibers increased with increasing CNT and PA6 content. The maximum value was achieved by 81.25 S/cm at P3-C20. The conductive network depended mainly on the connection conductivity among the carbon fiber, CNTs, and PA6 polymer matrix [21]. The homogeneous dispersion of CNTs between the carbon fibers with the polymer matrix leads to extensive overlap between the CNTs that establish three-dimensional conductive paths [20]. This mechanism was enhanced further as the content of CNTs and PA6 fiber increased. As a result, the electrical performance of the CNT–CF-nonwoven increased significantly with increasing CNT and PA6 short fiber content.

From the above results, it can be concluded that the conductive network composed of CNT–CF-nonwoven with PA6 short fibers may have some synergetic effect among the carbon fiber, CNTs, and PA6 matrix. Therefore, the transmission of electrons in the CNT–CF-nonwoven composite could be realized in three ways: (i) CF-to-CF, (ii) CNT-to-CNT, and (iii) CF-to-CNT. In addition, the PA6 short fibers provided a more rigid binding effect between the carbon fiber and CNTs. These are the main reasons for the observed positive effect of CNTs and PA6 on improving the electrical conductivities of the CNT–CF-nonwoven composites [24].

### 3.5. Thermal Properties of the CNT–CF-Nonwoven Composites

**Figure 7:**
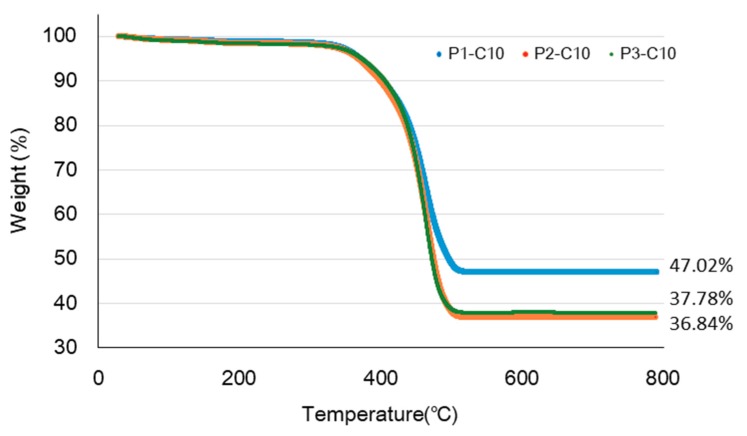
TGA curves of the CNT–CF-nonwoven composites according to the PA6 contents.

The thermal properties of the CNT–CF-nonwoven composites were examined by TGA to determine the effect of the PA6 contents on the thermal degradation of the composites, as shown in Figure 7. The samples, P1-C10, P2-C10, and P3-C10 exhibited similar thermal behavior. The weight loss in the initial process (~100 °C) was attributed to the removal of adsorbed water and decomposition of some oxygen-containing functional groups [24]. The weight losses in this temperature were 0.68%, 0.87%, and 1.02% for P1-10, P2-10, and P3-10, respectively. The curves show that thermal degradation began to occur only after the materials absorbed a certain amount of heat. The heat initiates the degradation process and breaks down the matrix structure of the material by cutting its molecular chain rupture or scission [13]. 

A sudden decrease in the mass of the composites occurred at approximately 350 °C, which indicated thermal degradation of PA6 [6]. The residual mass after degradation was possibly carbon fiber and CNTs. Owing to the addition of PA6 short fiber, the weight of the carbon fiber and CNTs decreased from 47% to 37%.

Despite the same content of carbon fiber and CNTs in the nonwoven composites, the mechanical properties and electrical conductivity tended to increase with increasing PA6 short fiber content. Therefore, the use of PA6 short fibers can impart high mechanical properties and electrical conductivity to the CNT–CF-nonwoven composite with the same CNT content compared to the CNT–CF-nonwoven without PA6.

## 4. Conclusions

Various types of carbon fiber-reinforced composites with improved mechanical properties and electrical conductivity were prepared with CNTs and PA6 short fibers using wet-laid and film stacking processes. 

The structure of the CNT–CF-nonwoven composite through wet-laid processing demonstrated more effective electrical conductivity when forming a single layer of a carbon fiber and CNT mixture, rather than forming carbon fiber and CNTs as individual layers. Therefore, the binding interaction between the carbon fiber and CNTs was increased by introducing PA6 short fibers into the carbon fiber and CNTs mixture to improve the mechanical properties of the nonwoven composite.

The morphology of the CNT–CF-nonwoven composites from SEM images show three-dimensionally distributed CNTs within the carbon fiber and polymer matrix. The mechanical properties of the nonwoven composites decreased with increasing CNT content only when CNTs were inserted. On the other hand, when CNTs and PA6 fiber were inserted simultaneously, the tensile strength increased with increasing CNT and PA6 fiber content because of the improved interfacial adhesion with matrix. The electrical conductivity of the composites was improved significantly with the addition of CNTs.

## Figures and Tables

**Table 1:** Properties of CNT–CF-nonwoven according to composite structure.

Characteristics	Thickness (mm)	Weight (g/m^2^)	Electrical Conductivity (S/cm)	Tensile Strength (MPa)	Modulus (MPa)
**PA6–CF–PA6**	0.288	181.6	27.75 ± 1.63	72.65 ± 12.46	3499 ± 275
**PA6–CF/CNT–PA6**	0.365	263.3	120.48 ± 21.14	46.21 ± 7.01	2946 ± 213
**PA6–CNT–PA6–CF–PA6**	0.325	305.0	33.11 ± 3.67	69.41 ± 14.66	3295 ± 539

**Table 2:** Preparations of the CNT–CF wet-laid composites.

Sample Code	CNT 0 wt %	CNT 1 wt %	CNT 5 wt %	CNT 10 wt %	CNT 20 wt %
**PA fiber 0** **wt** **%**	P1-C0	P1-C1	P1-C5	P1-C10	P1-C20
**PA fiber 50** **wt** **%**	P2-C0	P2-C1	P2-C5	P2-C10	P2-C20
**PA fiber 100** **wt** **%**	P3-C0	P3-C1	P3-C5	P3-C10	P3-C20

* PA6 and CNT: wt % with respect to carbon fiber. PA6 film for film stacking process: 58g/m^2^, 0.06 mm.
